# Discovery of novel and highly potent dual-targeting PKMYT1/HDAC2 inhibitors for hepatocellular carcinoma through structure-based virtual screening and biological evaluation

**DOI:** 10.3389/fphar.2024.1491497

**Published:** 2024-11-15

**Authors:** Yang Yang, Yuting Wang, Jing Chen, Miao-Miao Niu, Yongbin Wang, Xing Jin

**Affiliations:** ^1^ Department of Laboratory Medicine, The Affiliated Hospital of Yangzhou University, Yangzhou University, Yangzhou, China; ^2^ Department of Pharmaceutical Analysis, China Pharmaceutical University, Nanjing, China

**Keywords:** hepatocellular carcinoma, PKMYT1, HDAC2, dual-targeting inhibitors, virtual screening

## Abstract

Simultaneous inhibition of two or more pathways is playing a crucial role in the treatment of hepatocellular carcinoma with complex and diverse pathogenesis. However, there have been no reports of dual-targeting inhibitors for protein kinase membrane-associated tyrosine/threonine 1 (PKMYT1) and histone deacetylase 2 (HDAC2), which are critical targets for hepatocellular carcinoma treatment. Here, an integrated strategy of virtual screening was utilized to identify dual-targeting inhibitors for PKMYT1 and HDAC2. Notably, PKHD-5 has been identified as a potent inhibitor that selectively targets both PKMYT1 and HDAC2 with nanomolar affinity. Molecular dynamics have confirmed the strong binding stability of PKHD-5 with PKMYT1 and HDAC2. Importantly, it displayed a notably lower IC_50_ against the HepG2/MDR cell line, underscoring its potential to surmount drug resistance, while exhibiting minimal toxicity towards the normal liver cell line L02. Additionally, PKHD-5 has demonstrated significant antitumor proliferation effects without significant toxicity. In summary, the results suggest that PKHD-5 is a promising candidate for further preclinical studies of hepatocellular carcinoma therapy.

## 1 Introduction

Hepatocellular carcinoma (HCC), the predominant subtype of primary liver cancer, constitutes a major contributor to the global cancer burden and is the second most frequent cause of mortality associated with cancer worldwide (Llovet et al., 2022; Wang et al., 2020). Despite significant advances in the etiology of HCC, the 5-year survival rate of HCC patients was reported to be 5%–14% (Sarveazad et al., 2019). Therefore, treating HCC patients remains one of the most significant and increasingly challenging issues for global health (Singal et al., 2023). Despite the fact that sorafenib, a drug inhibiting several kinases, has obtained FDA approval for its utilization in the clinical management of HCC, the response rate was only 30%, and most of these patients developed resistance within 6 months (Llovet et al., 2015; Cheng et al., 2009; Gao et al., 2017). Thus, it is urgent to develop new targeted drugs for liver cancer to meet the needs of clinical treatment (Zhou et al., 2018).

The gene for protein kinase membrane-associated tyrosine/threonine 1 (PKMYT1), situated on the 16p13.3 band of chromosome 16 in humans, gives rise to the Myt1 kinase, an evolutionarily conserved protein kinase and a member of the WEE1 kinase family (Zhang et al., 2020; Najjar et al., 2019; Ghelli Luserna di Rorà et al., 2020). Functional PKMYT1 kinase phosphorylates Thr14 in the cyclin-dependent kinase (Cdk1)-cyclin B complex, retaining it in the cytoplasm to regulate the cell cycle (Najjar et al., 2019). PKMYT1 has exhibited to be overexpressed in various types of cancer, including hepatocellular carcinoma, as well as promoting tumor progression (Jeong et al., 2018; Agarwal et al., 2017).

**FIGURE 1:**
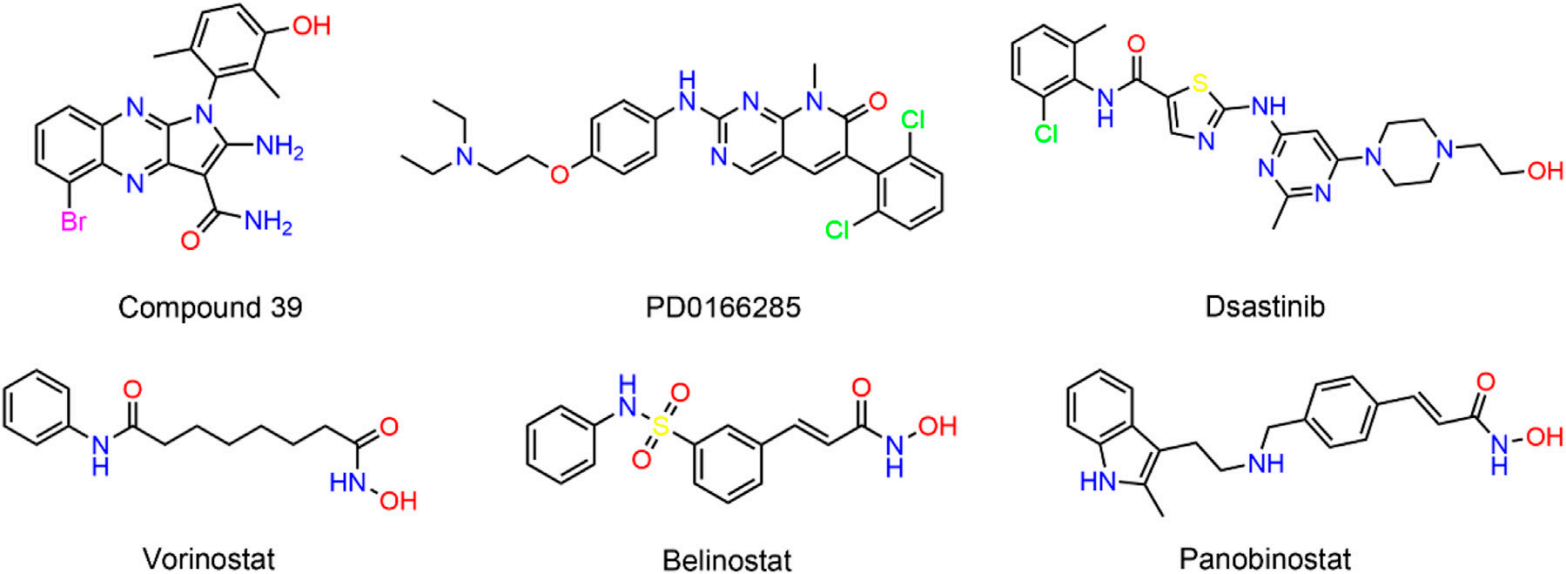
Representative structures of the reported inhibitors of PKMYT1 and HDAC2.

Researchers Liu and colleagues have demonstrated the oncogenic function of PKMYT1 in HCC, highlighting that the overexpression of PKMYT1 in HCC tumor samples stimulates cell growth and mobility by triggering the β-catenin/TCF signaling pathway (Liu et al., 2017). Wu et al. established HCC cell lines with PKMYT1 knockdown and found that PKMYT1 knockdown reduced the phosphorylation levels of p38 MAPK, ERK, and PI3K/Akt/mTOR, thereby inhibiting protective autophagy, inducing apoptosis, and ultimately inhibiting HCC progression (Wu et al., 2023). PKMYT1 is a compelling target for cancer therapy. However, few selective inhibitors targeting PKMYT1 have been reported (Schmidt et al., 2017). Szychowski et al. recently obtained the binding mode of compound 39 (Figure 1) to PKMYT1 through large-scale screening methods (Szychowski et al., 2022). The tyrosine kinase inhibitor dasatinib has exhibited inhibitory activity against PKMYT1 (Rohe et al., 2013). In addition, the pyrimidine-based molecule PD0166285 was recognized for its strong competitive binding to ATP sites. However, this agent exhibited a dual capability to suppress not only PKMYT1 but also the activity of the epidermal growth factor receptor (EGFR) and platelet-derived growth factor receptor (PDGFR), which could lead to severe toxic side effects (Hashimoto et al., 2006). Therefore, there is an immediate need for the development of structurally diverse, secure, and potent PKMYT1 inhibitors to fulfill the clinical treatment requirements of HCC patients.

Histone deacetylases (HDACs) modulate gene expression by detaching acetyl groups from particular lysine residues on the core histones (Haberland et al., 2009). To date, 18 HDAC homologs have been discovered, which could be subdivided into classes I, IIa, IIb, III, and IV (Wright and Menick, 2016). A growing body of evidence demonstrated that HDAC could be involved in tumorigenesis (Yoon and Eom, 2016; Ler et al., 2015). Particularly, HDAC2, a member of the class I HDAC family, was overexpressed in HCC tissues, and it was found that the expression level of HDAC2 was gradually upregulated with the increase of HCC histopathological grade (Nam et al., 2005). In addition, targeted interference with HDAC2 has been reported to reduce tumor cell growth and DNA synthesis in HCC cells (Noh et al., 2011). Moreover, Kim et al. demonstrated that epidermal growth factor (EGF) promoted HCC progression by inducing HDAC2 transcriptional activation in HCC cells via the CK2α/Akt signaling pathway (Kim et al., 2014). Although several HDAC inhibitors have been developed, such as Vorinostat (SAHA), Belinostat, and panobinostat, the HDAC inhibitors used to demonstrate cellular action are largely nonspecific to different HDAC isoforms, and clinical data indicated that these drugs possess disadvantages, such as limited efficacy and resistance (Sha et al., 2023; Lee et al., 2014; Chen et al., 2018).

To date, noticeable advancements have been achieved in unraveling the molecular pathogenesis of HCC, and it has been confirmed that genomic changes gradually alter the liver cell phenotype during the development of liver cancer, prompting further in-depth research into HCC treatment approaches based on epigenetics (Thorgeirsson and Grisham, 2002). Aberrant activation of the Wnt/β-catenin signaling pathway and overexpression of chromatin epigenetic factors are common in clinical tissues of liver cancer patients (Nath et al., 2015). Notably, some scholars have reported that the synergistic effect of WEE1 inhibitors and histone deacetylase inhibitors (HDACi) could block the DNA damage response (DDR), and WEE1 knockdown could significantly enhance the sensitivity of cancer cells to HDACi (Zhou et al., 2015). However, to our knowledge, there have been no reports to date of dual-targeting inhibitors for PKMYT1, a member of the WEE kinase family, and HDAC2 for the treatment of liver cancer.

Computer-aided drug design (CADD) technology has been applied in modern drug discovery and development, and docking-based virtual screening methods were frequently employed to identify new active compounds (Sabe et al., 2021; Valasani et al., 2014; Zheng et al., 2021). The identification of safe and potent dual-targeting PKMYT1/HDAC2 inhibitors by molecular docking-based virtual screening is a particularly important strategy for the treatment of liver cancer. Herein, we report a discovery of novel and highly potent dual-targeting PKMYT1/HDAC2 inhibitors through virtual screening and subsequent biological evaluation for the treatment of liver cancer.

## 2 Materials and methods

### 2.1 Materials

The L02, a cell strain indicative of normal hepatic function, and the Huh7, a strain indicative of HCC were both provided by Shanghai Institute of Cell Biology (Shanghai, China), and three other HCC cell lines (HepG2, Hep3B, and SNU449) were acquired from the American Type Culture Collection (ATCC; Manassas, VA, United States). Additionally, the multidrug-resistant HCC cell line (HepG2/MDR) was obtained from the Cell Bank of China Pharmaceutical University. HepG2, Hep3B, Huh7, and SNU449 lines were grown in a DMEM containing 10% fetal bovine serum (FBS), penicillin (100 IU/mL), and streptomycin (100 μg/mL) (Gibco, Grand Island, NY, United States). HepG2/MDR is maintained in RPMI 1640 medium containing 10% FBS and 50 μg/mL of penicillin/streptomycin. The cultured cells were kept and incubated under conditions of 37°C in a humidified atmosphere containing 5% CO_2_. All identified compounds were obtained from WuXi AppTec (Shanghai, China). Human recombinant PKMYT1 enzyme and HDAC2 enzyme were purchased from Abcam (Cambridge, United Kingdom).

### 2.2 Virtual screening

High-throughput computer virtual screening of compounds with potential activity was conducted as previously reported (Zhou et al., 2022). The crystallographic structures of PKMYT1 (PDB code: 8D6D) and HDAC2 (PDB code: 4LXZ) proteins were downloaded from the Protein Data Bank (PDB). The crystal structures were loaded into the Molecular Operating Environment (MOE, Chemical Computing Group Inc., Montreal, Quebec, Canada). The crystallographic structures were processed with the QuickPrep feature in MOE, a protocol that included the exclusion of non-bonded water molecules, the attachment of hydrogen atoms to polar groups, and the execution of energy optimization. A compound repository comprising 43,000 entities was synthesized through combinatorial chemistry techniques. Subsequently, the two-dimensional (2D) representations of all compounds within this repository were subjected to energy minimization to generate their corresponding three-dimensional (3D) conformations. The compounds were screened for molecular docking in MOE based on the crystal structures of PKMYT1 and HDAC2. The intermolecular interactions between receptor proteins and ligands were visualized and analyzed by applying the Ligand Interaction tool of MOE. Pocket shapes and interaction forces of ligand binding sites were analyzed by Surfaces and Maps tool. The selected compounds were docked to the active sites of PKMYT1 and HDAC2, respectively, using the Dock tool of MOE. Molecular docking was executed utilizing the Triangle Matcher protocol for the alignment of molecules and the London dG metric for assessing the energetics of the interactions. Compounds with the lowest docking scores were identified and selected for further analysis.

### 2.3 Enzyme inhibition assay

The impact of diverse compounds on the enzymatic activity of PKMYT1 was evaluated through the application of the ADP-GLO assay, as previously described (Szychowski et al., 2022). The ADP-GLO™ kinase assay kit, purchased from Promega Corporation (Madison, WI, United States), included the ADP-GLO reagent, the kinase assay reagent, and ultra-pure ATP and ADP. PKMYT1 enzyme was dissolved in 5 μL of freshly prepared enzyme assay buffer containing 70 mM Hepes, 3 mM MgCl_2_, 3 mM MnCl_2_, 50 μg/mL PEG20000, 3 μM sodium orthovanadate, 1.2 mM dithiothreitol, and then injected into 384-well plates. Next, the assay mixture was supplemented with the test compounds in DMSO, and then the solution was incubated for 15 min at room temperature. The enzymatic reaction was initiated by adding 5 μL of ATP, followed by a 60-min incubation at 30°C. Subsequently, 15 μL of ADP-GLO reagent was added, and the incubation was continued for an additional 40 min at room temperature. Then, 30 μL of kinase detection reagent was introduced, and the samples were incubated for a final 30 min. Luminescence measurements were taken using an Envision plate reader (PerkinElmer). The IC_50_ value of each tested compound was calculated by GraphPad Prism software.

The efficacy of the compound in inhibiting HDAC2 enzyme activity was determined following a protocol that was previously documented (Li et al., 2014). Firstly, a 96-well plate was loaded with a blend comprising 10 μL of HDAC2 enzyme solution and 50 μL of the test compounds at assorted concentrations, followed by incubation at a temperature of 37°C for a duration of 5 min. Subsequently, 10 μL of the fluorescent substrate Boc-Lys (acetyl)-AMC was added and continued for 30 min. Finally, 100 μL of developer containing trypsin and TSA was injected into each well and incubated for 20 min. The microplate reader (BioTek, United States) was utilized to quantify the fluorescence intensity at 390 nm and 460 nm wavelengths, and the IC_50_ values for each tested compound were calculated using GraphPad Prism software 6.0.

### 2.4 Kinase selectivity assays

The kinase selectivity analysis of PKHD-5 was performed using the SelectScreen kinase analysis service provided by Thermo Fisher Scientific. The experiment initially involved the preparation of a series of inhibitor solutions at varying concentrations, which were then reacted with various kinase targets under standardized conditions to calculate the percentage inhibition for each test solution. Ultimately, the IC_50_ values were calculated based on the concentration-inhibition curves by fitting a four-parameter logistic curve, thereby assessing the potency and selectivity of the inhibitors. The analysis results are detailed in Supplementary Table S1.

### 2.5 Molecular dynamics (MD) simulation

The crystal structures of PKMYT1 (PDB code: 8D6D) and HDAC2 (PDB code: 4LXZ) were obtained from the PDB and utilized as the initial coordinates for MD simulation. MD simulation was performed using GROMACS 2021.5 package under the AMBER99SB-ILDN force field with periodic boundary conditions. The complex was solvated in a 1.0 nm cubic simulation box using SPC water to maintain equilibrium. Na^+^ and Cl^−^ were added to neutralize the system. Afterwards, the solvated system was minimized with cutoff value of 50,000 steps. The NVT simulation was further carried out using a 100 ps V-rescale thermostat to keep the system’s temperature at 300 K. Subsequently, the Parrinello-Rahman barostat was utilized to conduct a 100 ps NPT simulation while keeping the system pressure at 1 bar. The DSSP program was used for protein secondary structure analysis. Lastly, the system underwent MD simulation for 50 ns, with trace data saved at intervals of 10 ps. Finally, GraphPad Prism 6.0 software was used to process the data and the root mean square deviation (RMSD), the root mean square fluctuation (RMSF) and protein secondary structure data were used to analyze the binding stability.

### 2.6 MTT assay

The MTT assay was conducted to evaluate the inhibitory impact of PKHD-5 on HCC cells, HepG2/ADR cells, and normal liver cells (Ding et al., 2020). Cells at a density of 4×10^3^ cells/well were inoculated into 96-well plates for overnight culture at 37°C. The cells were treated with varying concentrations of the test compounds and incubated for 48 h. Then, 100 μL of a 0.5 mg/mL MTT solution was pipetted into each well, and the incubation was prolonged for 4 h, resulting in the formation of a purple precipitate. Afterward, 200 μL of DMSO was added to each well, and the plate was vortexed for 10 min. The absorbance at 570 nm was determined with a Synergy 4 microplate reader (BioTek, United States). Each experiment was performed three times under the same conditions.

### 2.7 Western blot

To evaluate the protein levels of apoptosis-associated markers, we performed Western blot analysis on cell extracts as previously reported (Hao et al., 2018). Briefly, equal amounts of protein were subjected to SDS-PAGE and then transferred onto a polyvinylidene fluoride (PVDF) membrane. The membrane was incubated in a blocking solution consisting of 5% non-fat dry milk in Tris-buffered saline with Tween-20 (TBS-T) for 3 h at room temperature. Subsequently, the membrane was probed with primary antibodies specific to cleaved PARP and cleaved caspase-3 overnight at 4°C. After washing, the membrane was incubated with horseradish peroxidase-conjugated secondary antibodies for 1 h at room temperature. Immunoreactive bands were visualized using a chemiluminescent substrate (ECL, Pierce Technology). The relative expression levels of cleaved PARP and cleaved caspase-3 were quantified.

### 2.8 Real-time PCR (RT-PCR) analysis

Total RNA was extracted from cells using Trizol reagent (Invitrogen, Carlsbad, CA) and reverse transcribed into cDNA with the PrimeScript RT reagent kit (Takara Bio, Otsu, Japan). RT-PCR was performed on the Mx3000P system (Agilent Technologies, Santa Clara, CA) with TB Green Premix Ex Taq II (Takara Bio). Relative gene expression was determined by the 2^−ΔΔCt^ method.

### 2.9 HepG2 bearing animal model

A previously reported human tumor xenograft model was established to evaluate the anti-tumor effects of PKHD-5 *in vivo* (Zhou et al., 2022). All procedures involving animals were granted approval by the Ethics Committee at China Pharmaceutical University. A total of 20 male BALB/c nude mice (6 weeks old) were purchased from Changzhou Cave Experimental Animal Co., Ltd. (Changzhou, China). Mice were maintained under specific pathogen-free (SPF) conditions, with environmental parameters meticulously controlled at a temperature of 25°C ± 2°C, relative humidity maintained at 50% ± 5%, and an established diurnal cycle of 12 h for both light and darkness. During the experiment, all mice were free to access water and food. After 10 days of adaptive feeding, HepG2 cells (1×10^7^ cells) were subcutaneously injected into mice. Once the tumors reached a size of 90–120 mm^3^, the nude mice were randomly assigned to either the vehicle control group or the group treated with PKHD-5 (n = 5). Furthermore, the treatment was divided into three dose-based groups of 1, 5, and 10 mg/kg. The tumor volume was measured by a digital caliper for a total of 18 days and calculated by the following formula: (c×c×d)/2, where c represents the minimum diameter of the tumor and d indicates the maximum diameter. In addition, the body weight of the mice was measured and recorded every 3 days.

### 2.10 *In vivo* pharmacokinetics assessments

In the pharmacokinetic (PK) assessment of PKHD-5, BALB/c mice were utilized as the experimental model. PKHD-5 was administered either intraperitoneally or orally at a dosage of 5 mg/kg. Serial blood samples (0.25 mL) were collected at 0.25, 0.5, 1, 2, 4, 8, 16, and 24 h post-treatment. Each plasma sample (50 μL) underwent protein precipitation with a 1:1 mixture of methanol and acetonitrile (200 μL) and an internal standard. After vortexing for 5 min, the samples were centrifuged at 12,000 rpm for 5 min, yielding a supernatant (60 μL) for LC-MS/MS analysis. The data obtained were analyzed using Phoenix software to determine the PK profile of PKHD-5 in the mouse model.

## 3 Results and discussion

### 3.1 Virtual screening of PKMYT1/HDAC2 dual-targeting inhibitors

**FIGURE 2:**
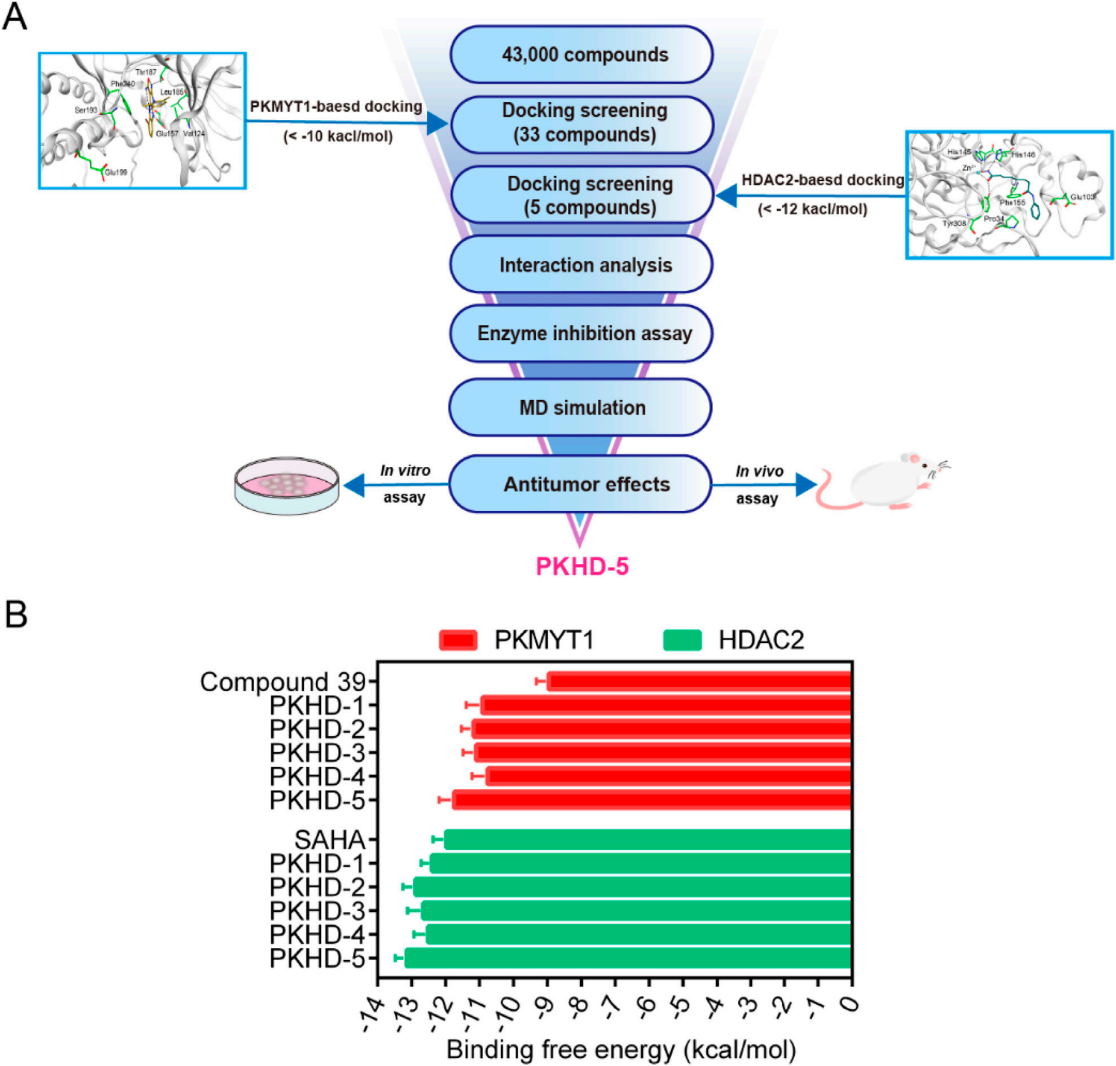
Virtual screening for dual PKMYT1/HDAC2-targeting compounds. **(A)** Flowchart of the integrated strategy of virtual screening and biological validation. **(B)** Binding free energy between PKHDs 1-5 and PKMYT1 and HDAC2, respectively.

**FIGURE 3:**
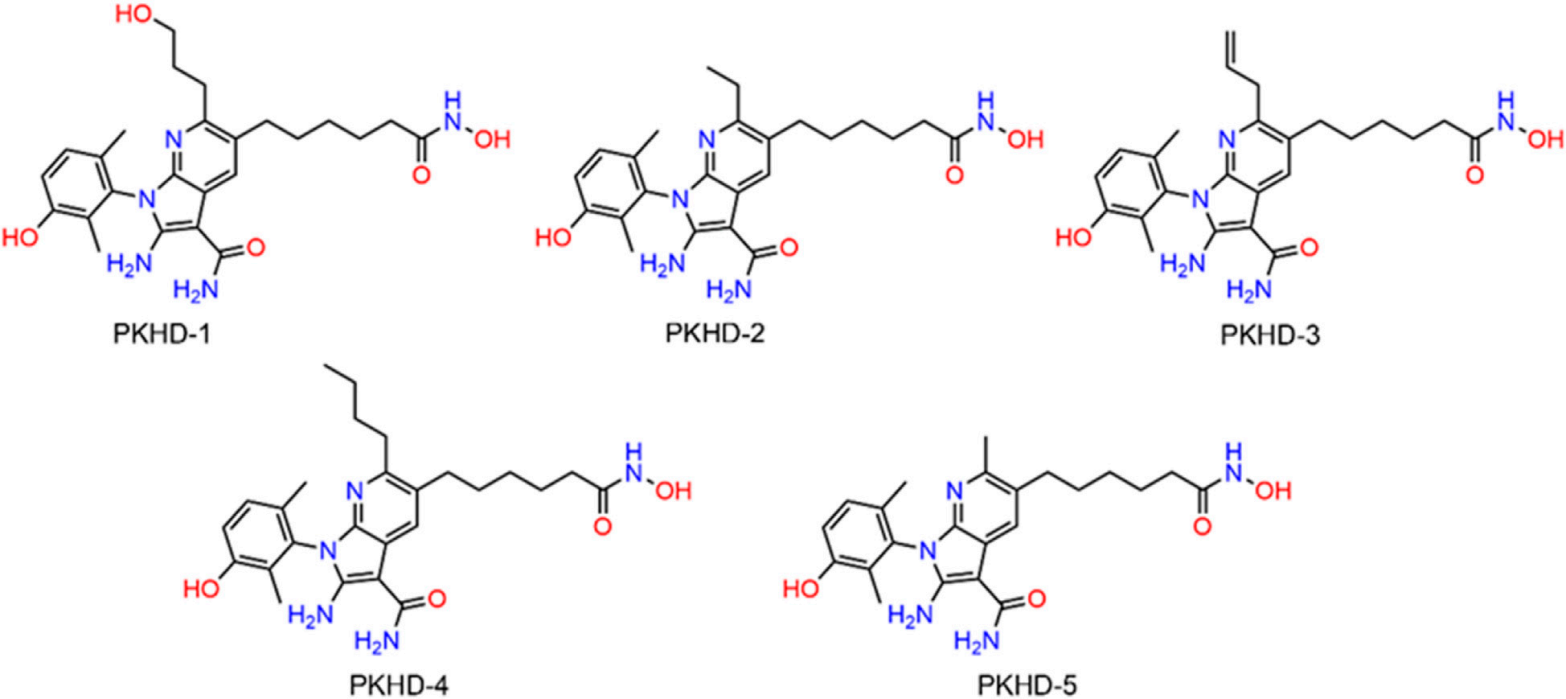
The structures of five hit compounds PKHDs 1-5 obtained through virtual screening.

The integrated strategy of virtual screening and biological validation is shown in Figure 2A. First, the 2D structures of 43,000 compounds from a small molecule database assembled by combinatorial chemistry methods were converted to 3D structures. Thereafter, based on the threshold value of the binding free energy of the initial ligand and PKMYT1, where lower binding free energy represents a higher affinity, the abovementioned compounds were first used for PKMYT1 docking screening, and 33 compounds below −10 kcal/mol were obtained. Next, using the binding free energy values of the original ligand and HDAC2 (−12 kcal/mol) as a reference, the obtained compounds were utilized in the docking screening of HDAC2. Ultimately, five compounds with binding free energy values lower than the reference ligand were identified and designated as PKHDs 1-5 (Figure 2B). The structures of PKHDs 1-5 are shown in Figure 3.

### 3.2 Interaction analysis

**FIGURE 4:**
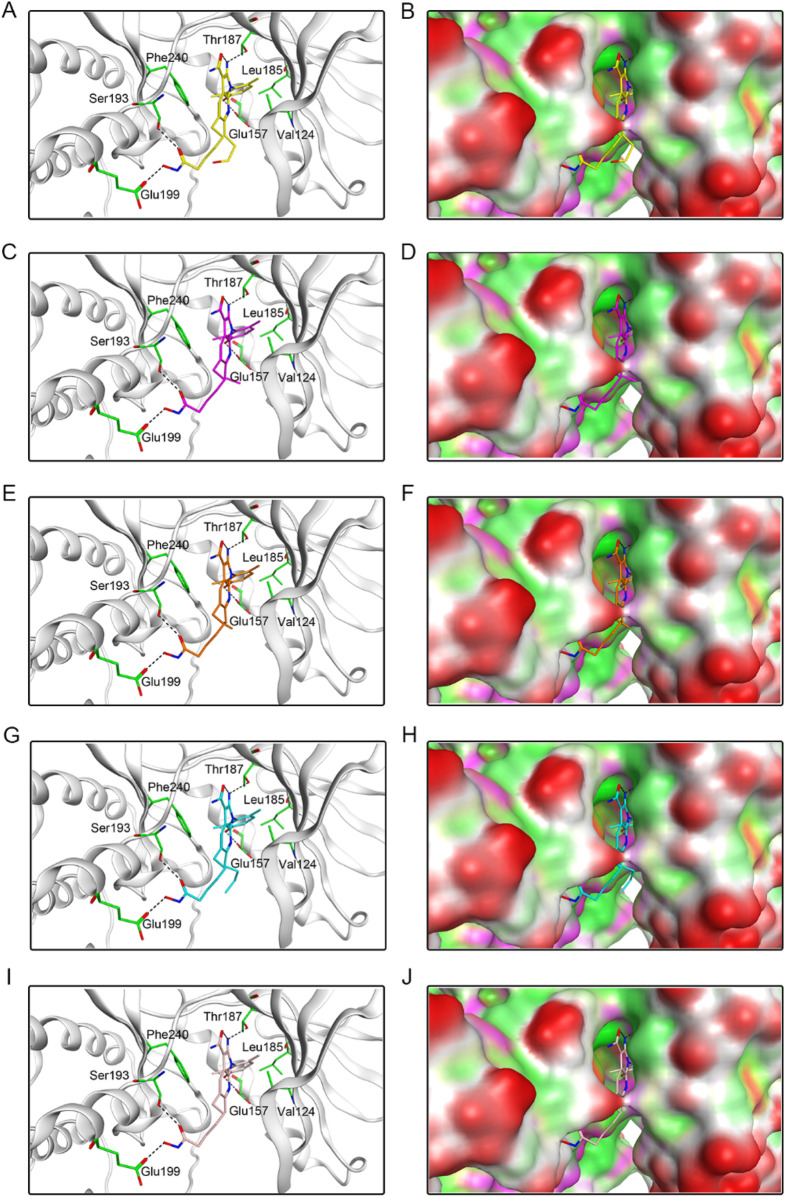
The binding modes of PKHDs 1-5 in the active site of PKMYT1. **(A, B)** The binding mode of PKHD-1(yellow sticks) in the active site of PKMYT1. **(C, D)** The binding mode of PKHD-2 (purple sticks) in the active site of PKMYT1. **(E, F)** The binding mode of PKHD-3 (orange sticks) in the active site of PKMYT1. **(G, H)** The binding mode of PKHD-4 (cyan sticks) in the active site of PKMYT1. **(I, J)** The binding mode of PKHD-5 (pink sticks) in the active site of PKMYT1. Residues in the active site are shown as green sticks. PKMYT1 is colored in light grey. The hydrogen bonds are presented in black dashed lines.

**FIGURE 5:**
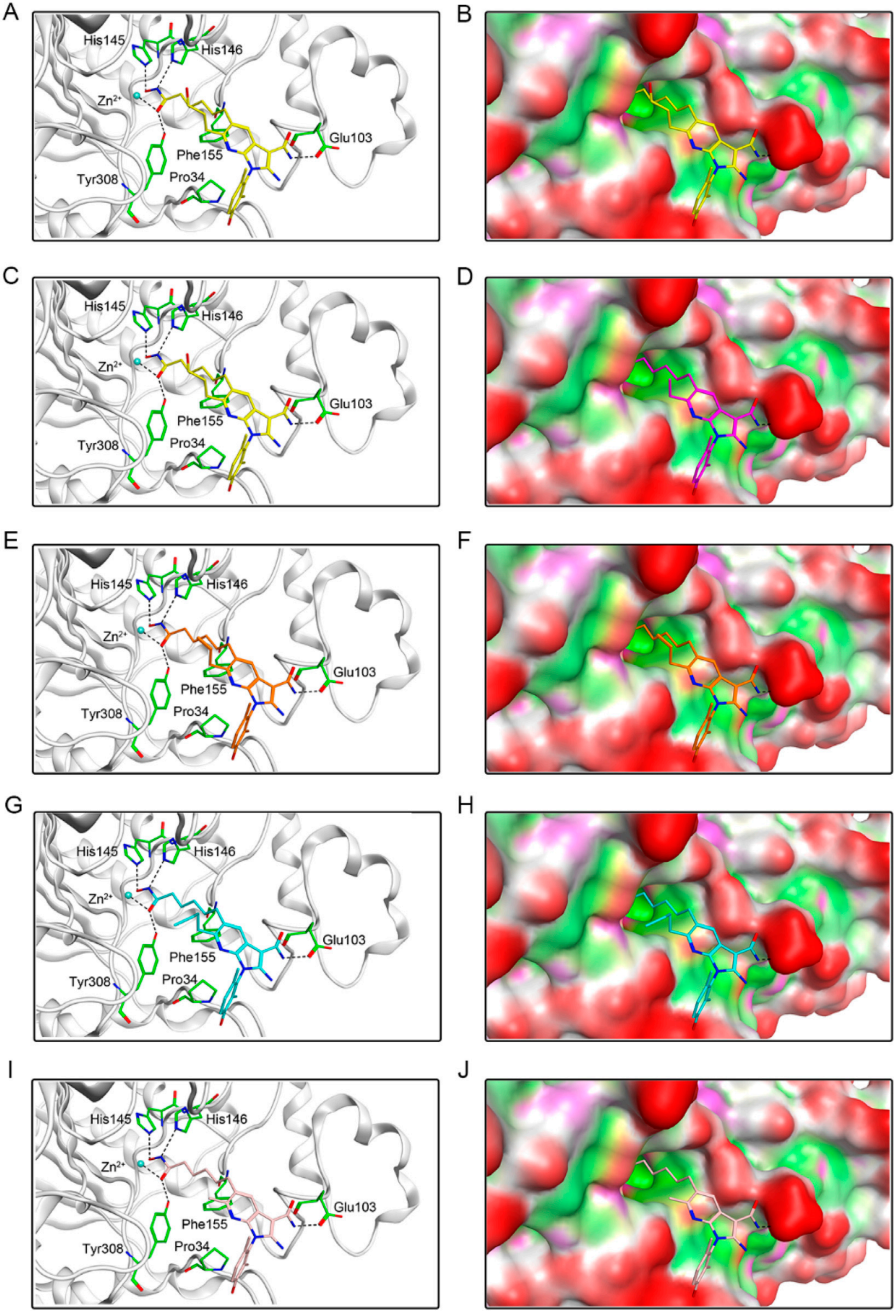
The binding modes of PKHDs 1-5 in the active site of HDAC2. **(A, B)** The binding mode of PKHD-1 (yellow sticks) in the active site of HDAC2. **(C, D)** The binding mode of PKHD-2 (purple sticks) in the active site of HDAC2. **(E, F)** The binding mode of PKHD-3 (orange sticks) in the active site of HDAC2. **(G, H)** The binding mode of PKHD-4 (cyan sticks) in the active site of HDAC2. **(I, J)** The binding mode of PKHD-5 (pink sticks) in the active site of HDAC2. The residues within the active site are depicted in green stick representation. HDAC2 is colored in light grey. Hydrogen bonds are illustrated with black dashed lines.

To investigate the interaction pattern of PKHDs 1-5 with their respective binding sites, molecular docking of PKMYT1 and HDAC2 was performed, respectively. Figure 4 displayed the binding modes and pocket surface maps of PKHDs 1-5 at the PKMYT1 active site. Each compound extends into the protein pocket, conforming perfectly to its shape. They formed important hydrogen bonding interactions with the side chains of amino acid residues inside the pocket, including Glu157, Thr187, Ser193, and Glu199. In addition, these compounds exerted hydrophobic interactions with Phe240, Leu185, and Val124 residues in the vicinity of the pocket, which stabilized the binding of the compounds to the receptor protein. The docking modes of PKHDs 1-5 with the HDAC2 are presented in the Figure 5. The carbon chains of the compounds extend into the interior of the HDAC2 pocket and the isohydroxamic acid group of each compound formed a critical ionic bond with the zinc ion and also formed hydrogen bonds with residues His146, His145, and Tyr308, which are essential for HDAC2 binding. Furthermore, each compound formed a hydrogen bond with residue Glu103 and hydrophobic interactions with residues Phe155 and Pro34. As illustrated in the surface plots, the compounds are well accommodated within the active pocket of HDAC2. Collectively, these results suggest that the PKHDs 1-5 can simultaneously interact with key residues of both PKMYT1 and HDAC2.

### 3.3 Highly selective inhibition of PKMYT1 and HDAC2 by PKHD-5

Five tested compounds obtained by molecular docking technology were further tested for their enzymatic inhibitory activity *in vitro*. The enzymatic inhibitory activity of PKHDs 1-5 on PKMYT1 and HDAC2 was measured by ADP-GLO method and fluorescence signal-based method, respectively. As shown in Table 1, the IC_50_ values of PKHDs 1-5 for PKMYT1 inhibition (IC_50_ = 3.15–13.56 nM) were significantly lower than that of compound 39 (IC_50_ = 15.48 ± 2.46 nM). The IC_50_ values of PKHDs 1-5 for HDAC2 inhibition (IC_50_ = 2.28–8.64 nM) were significantly lower than that by SAHA (IC_50_ = 10.09 ± 2.03 nM). In particular, PKHD-5 exhibited the greatest inhibitory activity on PKMYT1 and HDAC2, with a 4.91-fold and 4.42-fold inhibition of PKMYT1 and HDAC2 proteins, respectively, compared with the control compound 39 and SAHA. Furthermore, the compound PKHD-5 underwent a comprehensive kinase panel assay to assess its inhibitory potential across a broad spectrum of 63 distinct kinases. The data presented in Supplementary Table S1 reveal that PKHD-5 displayed IC_50_ values exceeding 50 μM for each of the 63 kinases evaluated. Collectively, these results underscore the remarkable selectivity of PKHD-5 for PKMYT1 and HDAC2, mitigating the risk of off-target effects and associated toxicity. Therefore, PKHD-5 was selected for the following molecular dynamics (MD) simulation and anti-tumor effects evaluation.

**TABLE 1:** Inhibitory activities of PKHDs 1-5, compound 39, and SAHA.

Compounds	PKMYT1 (IC_50_, nM)	HDAC2 (IC_50_, nM)
PKHD-1	11.09 ± 1.86	8.64 ± 1.35
PKHD-2	7.78 ± 0.91	4.14 ± 0.52
PKHD-3	9.26 ± 1.07	5.47 ± 0.66
PKHD-4	13.56 ± 1.94	7.31 ± 1.12
PKHD-5	3.15 ± 0.21	2.28 ± 0.13
Compound 39	15.48 ± 2.46	no inhibition
SAHA	no inhibition	10.09 ± 2.03

### 3.4 MD simulation

**FIGURE 6:**
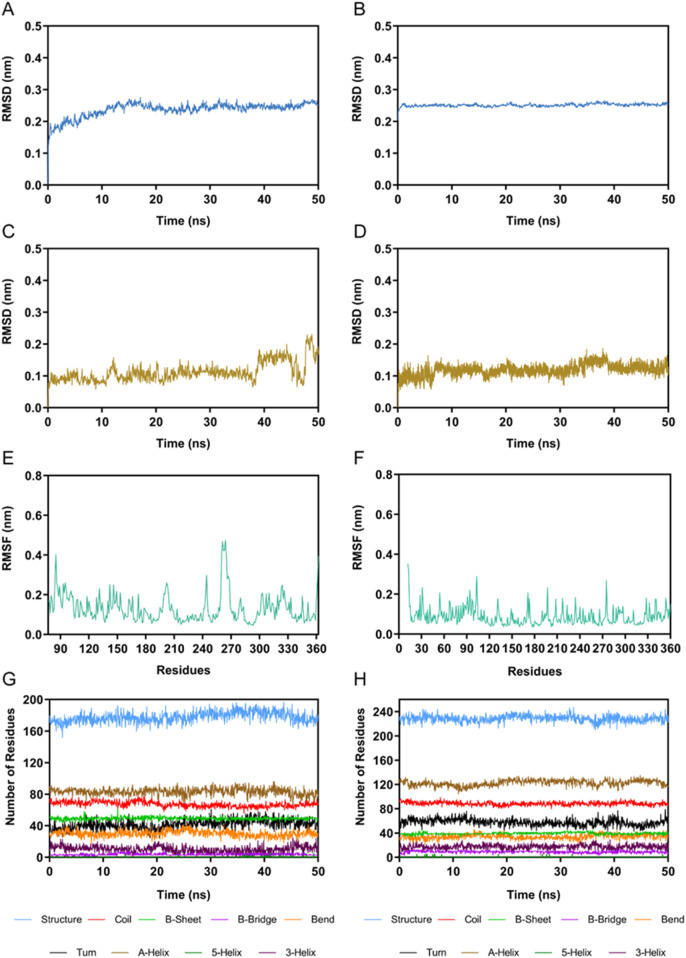
MD simulation of PKHD-5 in complex of PKMYT1 and HDAC2. **(A)** RMSD analysis of the PKMYT1-PKHD-5 complex. **(B)** RMSD analysis of the HDAC2-PKHD-5 complex. **(C)** The RMSD of PKHD-5 atoms within the PKMYT1-PKHD-5 complex. **(D)** The RMSD of PKHD-5 atoms within the HDAC2-PKHD-5 complex. **(E)** RMSF analysis of PKMYT1. **(F)** RMSF analysis of HDAC2. **(G, H)** The secondary structures analysis of PKMYT1 and HDAC2, respectively.

The PKMYT1-PKHD-5 and HDAC2-PKHD-5 complexes were further assessed by 100 ns MD simulation to evaluate the temporally evolving binding interactions, structural robustness, and conformational fluctuations. The RMSD of PKMYT1-PKHD-5 complex was initially risen and eventually stabilized between about 0.2 nm and 0.3 nm (Figure 6A). The fluctuations in the RMSD of HDAC2-PKHD-5 complex were extremely small and stabilized at approximately 0.25 nm (Figure 6B). Moreover, the dynamic stability of PKHD-5 was also explored in two protein complexes, respectively. As illustrated in Figures 6C, D, the range of RMSD values of PKHD-5 remained almost below 0.2 nm during binding to PKMYT1, except for a slight fluctuation after 40 ns. In the HDAC2 complex, the RMSD values of PKHD-5 were below 0.2 nm, demonstrating dynamic binding stability to HDAC2 protein. Furthermore, during the 50 ns MD simulation, the residual flexibility of PKMYT1 and HDAC2 was assessed by analyzing the RMSF of all atoms. As depicted in Figures 6E, F, the key residues of PKMYT1 and HDAC2 had small fluctuations throughout the simulation process. In addition, Further analysis using the DSSP method was performed to investigate changes in the secondary structural compositions of PKMYT1 and HDAC2. As illustrated in Figures 6G, H, the results of molecular docking and MD simulation indicated that PKHD-5 could interact with PKMYT1 and HDAC2 critical site residues with notable binding stability.

### 3.5 *In vitro* anti-tumor effect

The cytotoxic effects of PKHD-5 were evaluated using the MTT assay on a panel of HCC cell lines, including HepG2, Hep3B, Huh7, and SNU449, as well as the multidrug-resistant HepG2/MDR cell line and the normal human liver cell line L02. As shown in Table 2, PKHD-5 exhibited strong cytotoxicity against all HCC cell lines with IC_50_ values ranging from 0.19 to 0.92 μM, with the highest inhibitory potency observed in HepG2 cells (IC_50_ = 0.19 ± 0.03 μM). Notably, PKHD-5 also showed a significantly lower IC_50_ value in the HepG2/MDR cell line (IC_50_ = 0.92 ± 0.05 μM) compared to Compound 39 (IC_50_ > 100 μM) and SAHA (IC_50_ = 6.84 ± 0.52 μM), suggesting its potential to overcome drug resistance. Moreover, PKHD-5 displayed minimal toxicity towards the normal liver cell line L02 (IC_50_ > 100 μM), which is a significant advantage over Compound 39 (IC_50_ = 11.73 ± 0.82 μM) and SAHA (IC_50_ = 79.02 ± 6.31 μM).

**TABLE 2:** *In vitro* cytotoxicity of PKHD-5, compound 39, and SAHA against liver cancer cells and normal cells after 48 h assessed by MTT assay.

Name	IC_50_ (μM) ± SD^a^					
	HepG2	Hep3B	Huh7	SNU449	HepG2/MDR	L02
PKHD-5	0.19 ± 0.03	0.38 ± 0.05	0.47 ± 0.04	0.59 ± 0.06	0.92 ± 0.05	>100
Compound 39	1.03 ± 0.07	1.16 ± 0.09	1.36 ± 0.14	1.52 ± 0.13	>100	11.73 ± 0.82
SAHA	0.98 ± 0.06	1.25 ± 0.11	1.43 ± 0.08	2.07 ± 0.22	6.84 ± 0.52	79.02 ± 6.31

^a^ Each experiment was performed at least three times. Data were presented as the mean ± SD.

### 3.6 Apoptosis induction and molecular effects of PKHD-5

**FIGURE 7:**
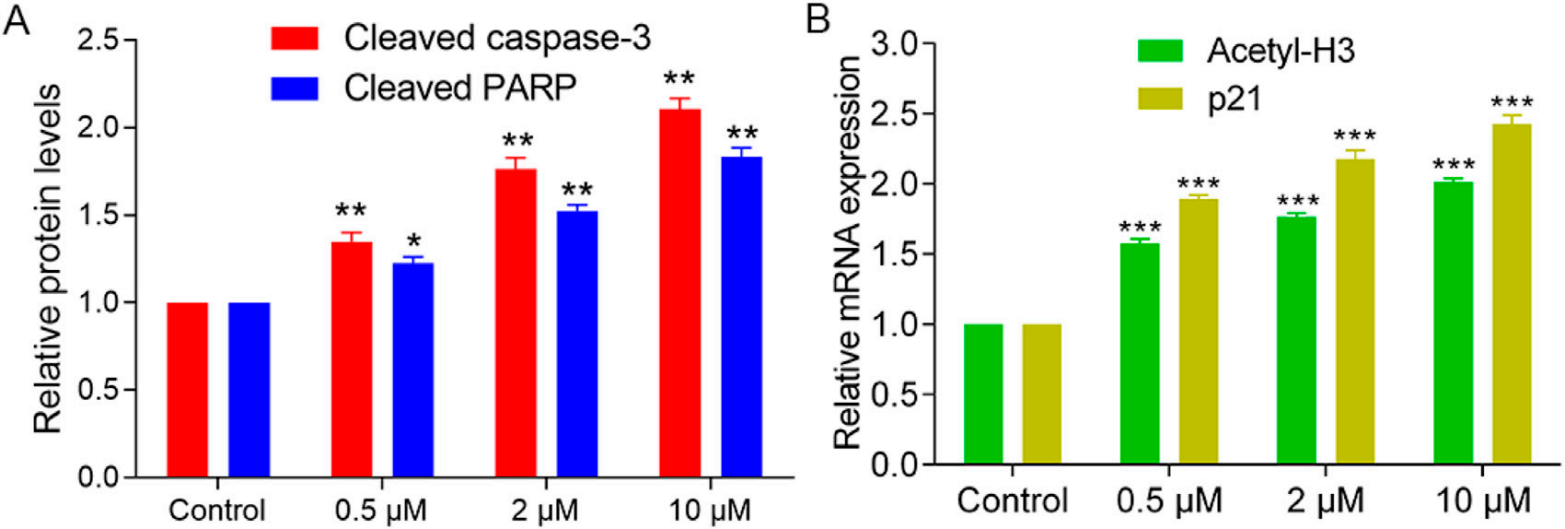
Effects of PKHD-5 on HepG2 cells. **(A)** Western blot analysis showing the impact of PKHD-5 on the levels of cleaved caspase-3 and cleaved PARP in HepG2 cells. **(B)** RT-PCR analysis demonstrating the influence of PKHD-5 on histone H3 acetylation and p21 expression in HepG2 cells. Significance levels of **p < 0.01 and ***p < 0.001 are used to denote statistically significant differences from the control group.

Given the selective cytotoxicity of PKHD-5 observed in HepG2 cells as per our MTT assay, we further investigated the molecular effects of PKHD-5. Western blot analysis (Figure 7A) indicated that PKHD-5 significantly induced apoptosis, as evidenced by increased levels of cleaved caspase-3 and cleaved PARP. Furthermore, RT-PCR analysis (Figure 7B) revealed that PKHD-5 upregulated the expression of histone H3 acetylation, a hallmark of HDAC2 inhibition, and p21, which is involved in cell cycle arrest and is influenced by both PKMYT1 and HDAC2 activities (Wang et al., 2024; Goder et al., 2018). This suggests that PKHD-5 may modulate the activities of these enzymes, thereby affecting gene expression and cell cycle regulation. These findings support the potential of PKHD-5 as an anticancer agent and warrant further investigation into its mechanisms of action.

### 3.7 *In vivo* anti-tumor effect

**FIGURE 8:**
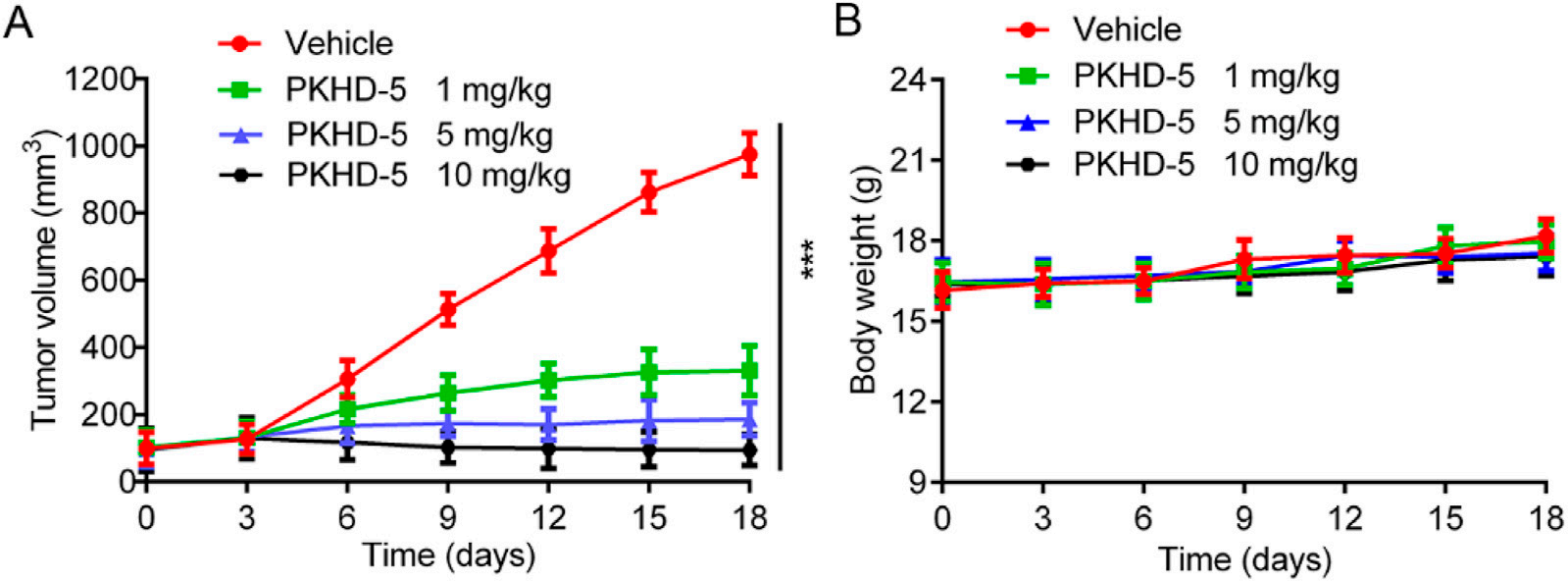
*In vivo* antitumor activity of PKHD-5. **(A)** The changes in tumor volume with varying concentrations of PKHD-5. **(B)** Body weight changes in mice. The results are presented as mean ± SD. ***p < 0.001 denotes a statistically significant difference compared to the control group.

Given that PKHD-5 exhibited the strongest inhibitory effect on PKMYT1 and HDAC2 enzymes *in vitro* and significantly inhibited the proliferation of HepG2 cells, the effect of PKHD-5 on xenograft mice bearing the HepG2 cell line was further assessed. A vehicle control and different doses of PKHD-5 were injected intraperitoneally into BALB/c nude mice. As illustrated in Figure 8A, the rate at which tumors grew in the group treated with PKHD-5 was considerably lower after 18 days of treatment, showing a clear dose-dependent reduction when compared to the vehicle control group. Notably, the greatest anti-tumor effect was observed at a dose of 10 mg/kg administered. Furthermore, the body weight changes in mice did not exhibit any significant disparities between the group administered PKHD-5 and the group given the vehicle (Figure 8B). Therefore, the results of *in vivo* anti-tumor effect assay indicated that PKHD-5 had a strong anti-tumor efficacy without obvious side effects, suggesting its potential as a viable therapeutic agent for HCC.

### 3.8 *In vivo* PK evaluation of PKHD-5

Following a single intraperitoneal administration of 5 mg/kg in BALB/c mice, the PK parameters of PKHD-5 were presented in Supplementary Table S2. The terminal half-life (T_1/2_) of PKHD-5 was determined to be 4.75 h, suggesting a relatively rapid clearance from the systemic circulation. The maximum concentration (Cmax) of PKHD-5 reached 3264 nmol·mL^-1^, reflecting the peak drug exposure shortly after administration. The area under the curve (AUC) value was 15,041 h·nmol·mL^-1^, indicating that PKHD-5 provided significant drug exposure over the dosing interval, potentially correlating with its pharmacodynamic effects. The bioavailability (F%) of PKHD-5 was found to be 82%, suggesting that a substantial portion of the administered dose was absorbed into the systemic circulation. Collectively, these data indicated that PKHD-5 exhibited a balanced profile between systemic exposure and elimination, with high bioavailability suggesting potential for effective oral dosing. Consequently, we conducted further PK testing following oral administration of PKHD-5 at 5 mg/kg, the results of which are presented in Supplementary Table S2. The T1/2 of PKHD-5 was determined to be 3.93 h, which is slightly shorter than that observed following intraperitoneal injection, potentially due to the first-pass effect associated with oral administration. The Cmax was 2915 nmol·mL^-1^, the AUC was 12,163 h·nmol·mL^-1^, and the bioavailability (F%) was 66%, indicating a satisfactory oral bioavailability. These data suggest that PKHD-5 provides effective systemic exposure following both intraperitoneal injection and oral administration, with the oral route demonstrating a high bioavailability, supporting its potential as a candidate for oral dosing. This pharmacokinetic profile lays the groundwork for further investigation into the clinical potential of PKHD-5.

## 4 Conclusion

The development of dual-targeted small molecule inhibitors has garnered significant attention in the field of oncology due to their enhanced efficacy, favorable safety profiles, and improved cell permeability. Despite the approval of sorafenib, a multikinase inhibitor, its clinical utility is limited by suboptimal efficacy and the emergence of drug resistance, often due to the activation of alternative signaling pathways.

In this study, we employed a rational drug design approach, utilizing docking-based virtual screening and interaction analysis, to identify a series of small molecules with dual-targeting capabilities against PKMYT1 and HDAC2. These proteins are overexpressed in hepatocellular carcinoma (HCC) tissues and are implicated in the promotion of tumorigenesis and progression. Among the identified inhibitors, PKHD-5 demonstrated the most potent inhibitory effects on both PKMYT1 (IC_50_ = 3.15 ± 0.21 nM) and HDAC2 (IC_50_ = 2.28 ± 0.13 nM). Kinase selectivity profiling further validated PKHD-5’s specificity for these targets, which is essential for reducing potential off-target effects and toxicity in HCC treatment. *In vitro* studies demonstrated that PKHD-5 significantly suppressed tumor cell viability and proliferation in HCC cell lines, most notably in HepG2 cells. Importantly, PKHD-5 had a minimal impact on normal liver cells (L02), indicating a selective toxicity that is beneficial for reducing side effects and preserving the health of non-cancerous liver cells. This selective action is a critical advantage in the development of targeted cancer therapies. Furthermore, the safety profile of PKHD-5 was superior to that of existing inhibitors, as evidenced by its higher IC_50_ value against normal liver cells compared to compound 39 and SAHA. This finding is particularly relevant given the need for HCC treatments with a favorable therapeutic index to mitigate the risk of hepatotoxicity and other adverse effects. *In vivo* studies using a xenograft model further substantiated the anti-tumor efficacy of PKHD-5, with significant tumor growth inhibition observed without concurrent significant side effects. These findings underscore the potential of PKHD-5 as a promising candidate for the therapeutic intervention of HCC.

In conclusion, the dual-targeting small molecule PKHD-5, which inhibits both PKMYT1 and HDAC2, emerges as a promising candidate for HCC therapy. Its multi-pathway targeting approach, potent inhibitory effects, and favorable safety profile offer distinct advantages over current treatments, addressing the critical unmet needs of enhanced efficacy and reduced resistance. Future preclinical studies will be directed towards elucidating the mechanistic underpinnings of PKHD-5’s action, optimizing its pharmacokinetic properties, and identifying predictive biomarkers of response to guide its clinical development. Considering the complexity of kinase and HDAC networks within cells, to further mitigate potential off-target effects, we will conduct long-term toxicity studies in the future to monitor any chronic side effects that PKHD-5 may induce, ensuring its safety.

## Data Availability

The original contributions presented in the study are included in the article/Supplementary Material, further inquiries can be directed to the corresponding author.

## References

[B1] Agarwal R. Narayan J. Bhattacharyya A. Saraswat M. Tomar A. K. (2017). Gene expression profiling, pathway analysis and subtype classification reveal molecular heterogeneity in hepatocellular carcinoma and suggest subtype specific therapeutic targets. Cancer Genet. 216-217, 37–51. 10.1016/j.cancergen.2017.06.002 29025594

[B2] Chen D. Soh C. K. Goh W. H. Wang H. (2018). Design, synthesis, and preclinical evaluation of fused pyrimidine-based hydroxamates for the treatment of hepatocellular carcinoma. J. Med. Chem. 61, 1552–1575. 10.1021/acs.jmedchem.7b01465 29360358

[B3] Cheng A. L. Kang Y. K. Chen Z. Tsao C. J. Qin S. Kim J. S. (2009). Efficacy and safety of sorafenib in patients in the Asia-Pacific region with advanced hepatocellular carcinoma: a phase III randomised, double-blind, placebo-controlled trial. Lancet Oncol. 10, 25–34. 10.1016/S1470-2045(08)70285-7 19095497

[B4] Ding L. Y. Hou Y. C. Kuo I. Y. Hsu T. Y. Tsai T. C. Chang H. W. (2020). Epigenetic silencing of AATK in acinar to ductal metaplasia in murine model of pancreatic cancer. Clin. Epigenetics 12, 87. 10.1186/s13148-020-00878-6 32552862 PMC7301993

[B5] Gao L. Wang X. Tang Y. Huang S. Hu C. A. Teng Y. (2017). FGF19/FGFR4 signaling contributes to the resistance of hepatocellular carcinoma to sorafenib. J. Exp. Clin. Cancer Res. 36, 8. 10.1186/s13046-016-0478-9 28069043 PMC5223586

[B6] Ghelli Luserna di Rorà A. Cerchione C. Martinelli G. Simonetti G. (2020). A WEE1 family business: regulation of mitosis, cancer progression, and therapeutic target. J. Hematol. & Oncol. 13, 126. 10.1186/s13045-020-00959-2 32958072 PMC7507691

[B7] Goder A. Emmerich C. Nikolova T. Kiweler N. Schreiber M. Kuhl T. (2018). HDAC1 and HDAC2 integrate checkpoint kinase phosphorylation and cell fate through the phosphatase-2A subunit PR130. Nat. Commun. 9, 764. 10.1038/s41467-018-03096-0 29472538 PMC5823910

[B8] Haberland M. Montgomery R. L. Olson E. N. (2009). The many roles of histone deacetylases in development and physiology: implications for disease and therapy. Nat. Rev. Genet. 10, 32–42. 10.1038/nrg2485 19065135 PMC3215088

[B9] Hao C. Zhao F. Song H. Guo J. Li X. Jiang X. (2018). Structure-based design of 6-Chloro-4-aminoquinazoline-2-carboxamide derivatives as potent and selective p21-activated kinase 4 (PAK4) inhibitors. J. Med. Chem. 61, 265–285. 10.1021/acs.jmedchem.7b01342 29190083

[B10] Hashimoto O. Shinkawa M. Torimura T. Nakamura T. Selvendiran K. Sakamoto M. (2006). Cell cycle regulation by the Wee1 inhibitor PD0166285, pyrido [2,3-d] pyimidine, in the B16 mouse melanoma cell line. BMC Cancer 6, 292. 10.1186/1471-2407-6-292 17177986 PMC1770931

[B11] Jeong D. Kim H. Kim D. Ban S. Oh S. Ji S. (2018). Protein kinase, membrane‑associated tyrosine/threonine 1 is associated with the progression of colorectal cancer. Oncol. Rep. 39, 2829–2836. 10.3892/or.2018.6371 29658598

[B12] Kim H. S. Chang Y. G. Bae H. J. Eun J. W. Shen Q. Park S. J. (2014). Oncogenic potential of CK2α and its regulatory role in EGF-induced HDAC2 expression in human liver cancer. FEBS J. 281, 851–861. 10.1111/febs.12652 24616922

[B13] Lee Y.-H. Seo D. Choi K.-J. Andersen J. B. Won M.-A. Kitade M. (2014). Antitumor effects in hepatocarcinoma of isoform-selective inhibition of HDAC2. Cancer Res. 74, 4752–4761. 10.1158/0008-5472.CAN-13-3531 24958469 PMC4155016

[B14] Ler S. Y. Leung C. H. Khin L. W. Lu G. D. Salto-Tellez M. Hartman M. (2015). HDAC1 and HDAC2 independently predict mortality in hepatocellular carcinoma by a competing risk regression model in a Southeast Asian population. Oncol. Rep. 34, 2238–2250. 10.3892/or.2015.4263 26352599 PMC4583520

[B15] Li X. Inks E. S. Li X. Hou J. Chou C. J. Zhang J. (2014). Discovery of the first N-hydroxycinnamamide-based histone deacetylase 1/3 dual inhibitors with potent oral antitumor activity. J. Med. Chem. 57, 3324–3341. 10.1021/jm401877m 24694055 PMC4030833

[B16] Liu L. Wu J. Wang S. Luo X. Du Y. Huang D. (2017). PKMYT1 promoted the growth and motility of hepatocellular carcinoma cells by activating beta-catenin/TCF signaling. Exp. Cell Res. 358, 209–216. 10.1016/j.yexcr.2017.06.014 28648520

[B17] Llovet J. M. Castet F. Heikenwalder M. Maini M. K. Mazzaferro V. Pinato D. J. (2022). Immunotherapies for hepatocellular carcinoma. Nat. Rev. Clin. Oncol. 19, 151–172. 10.1038/s41571-021-00573-2 34764464

[B18] Llovet J. M. Villanueva A. Lachenmayer A. Finn R. S. (2015). Advances in targeted therapies for hepatocellular carcinoma in the genomic era. Nat. Rev. Clin. Oncol. 12, 408–424. 10.1038/nrclinonc.2015.103 26054909

[B19] Najjar A. Platzer C. Luft A. Aßmann C. A. Elghazawy N. H. Erdmann F. (2019). Computer-aided design, synthesis and biological characterization of novel inhibitors for PKMYT1. Eur. J. Med. Chem. 161, 479–492. 10.1016/j.ejmech.2018.10.050 30388464

[B20] Nam S. W. Park J. Y. Ramasamy A. Shevade S. Islam A. Long P. M. (2005). Molecular changes from dysplastic nodule to hepatocellular carcinoma through gene expression profiling. Hepatology 42, 809–818. 10.1002/hep.20878 16175600

[B21] Nath A. Li I. Roberts L. R. Chan C. (2015). Elevated free fatty acid uptake via CD36 promotes epithelial-mesenchymal transition in hepatocellular carcinoma. Sci. Rep. 5, 14752. 10.1038/srep14752 26424075 PMC4589791

[B22] Noh J. H. Jung K. H. Kim J. K. Eun J. W. Bae H. J. Xie H. J. (2011). Aberrant regulation of HDAC2 mediates proliferation of hepatocellular carcinoma cells by deregulating expression of G1/S cell cycle proteins. PLoS One 6, e28103. 10.1371/journal.pone.0028103 22132221 PMC3223227

[B23] Rohe A. Göllner C. Wichapong K. Erdmann F. Al-Mazaideh G. M. A. Sippl W. (2013). Evaluation of potential Myt1 kinase inhibitors by TR-FRET based binding assay. Eur. J. Med. Chem. 61, 41–48. 10.1016/j.ejmech.2012.06.007 22770610

[B24] Sabe V. T. Ntombela T. Jhamba L. A. Maguire G. E. M. Govender T. Naicker T. (2021). Current trends in computer aided drug design and a highlight of drugs discovered via computational techniques: a review. Eur. J. Med. Chem. 224, 113705. 10.1016/j.ejmech.2021.113705 34303871

[B25] Sarveazad A. Agah S. Babahajian A. Amini N. Bahardoust M. (2019). Predictors of 5 year survival rate in hepatocellular carcinoma patients. J. Res. Med. Sci. 24, 86. 10.4103/jrms.JRMS_1017_18 31741658 PMC6856560

[B26] Schmidt M. Rohe A. Platzer C. Najjar A. Erdmann F. Sippl W. (2017). Regulation of G2/M transition by inhibition of WEE1 and PKMYT1 kinases. Molecules 22, 2045. 10.3390/molecules22122045 29168755 PMC6149964

[B27] Sha Y. Pan M. Chen Y. Qiao L. Zhou H. Liu D. (2023). PLEKHG5 is stabilized by HDAC2-related deacetylation and confers sorafenib resistance in hepatocellular carcinoma. Cell Death Discov. 9, 176. 10.1038/s41420-023-01469-z 37248230 PMC10227013

[B28] Singal A. G. Kanwal F. Llovet J. M. (2023). Global trends in hepatocellular carcinoma epidemiology: implications for screening, prevention and therapy. Nat. Rev. Clin. Oncol. 20, 864–884. 10.1038/s41571-023-00825-3 37884736

[B29] Szychowski J. Papp R. Dietrich E. Liu B. Vallee F. Leclaire M. E. (2022). Discovery of an orally bioavailable and selective PKMYT1 inhibitor, RP-6306. J. Med. Chem. 65, 10251–10284. 10.1021/acs.jmedchem.2c00552 35880755 PMC9837800

[B30] Thorgeirsson S. S. Grisham J. W. (2002). Molecular pathogenesis of human hepatocellular carcinoma. Nat. Genet. 31, 339–346. 10.1038/ng0802-339 12149612

[B31] Valasani K. R. Vangavaragu J. R. Day V. W. Yan S. S. (2014). Structure based design, synthesis, pharmacophore modeling, virtual screening, and molecular docking studies for identification of novel cyclophilin D inhibitors. J. Chem. Inf. Model 54, 902–912. 10.1021/ci5000196 24555519 PMC3985759

[B32] Wang M. Liao J. Wang J. Xu M. Cheng Y. Wei L. (2024). HDAC2 promotes autophagy-associated HCC malignant progression by transcriptionally activating LAPTM4B. Cell Death Dis. 15, 593. 10.1038/s41419-024-06981-3 39147759 PMC11327261

[B33] Wang X. H. Long L. H. Cui Y. Jia A. Y. Zhu X. G. Wang H. Z. (2020). MRI-based radiomics model for preoperative prediction of 5-year survival in patients with hepatocellular carcinoma. Br. J. Cancer 122, 978–985. 10.1038/s41416-019-0706-0 31937925 PMC7109104

[B34] Wright L. H. Menick D. R. (2016). A class of their own: exploring the nondeacetylase roles of class IIa HDACs in cardiovascular disease. Am. J. Physiol. Heart Circ. Physiol. 311, H199–H206. 10.1152/ajpheart.00271.2016 27208161 PMC5005290

[B35] Wu F. Tu C. Zhang K. Che H. Lin Q. Li Z. (2023). Knockdown of PKMYT1 is associated with autophagy inhibition and apoptosis induction and suppresses tumor progression in hepatocellular carcinoma. Biochem. Biophys. Res. Commun. 640, 173–182. 10.1016/j.bbrc.2022.11.084 36512849

[B36] Yoon S. Eom G. H. (2016). HDAC and HDAC inhibitor: from cancer to cardiovascular diseases. Chonnam Med. J. 52, 1–11. 10.4068/cmj.2016.52.1.1 26865995 PMC4742605

[B37] Zhang Q.-Y. Chen X.-Q. Liu X.-C. Wu D.-M. (2020). PKMYT1 promotes gastric cancer cell proliferation and apoptosis resistance. OncoTargets Ther. Vol. 13, 7747–7757. 10.2147/ott.s255746 PMC7414979 32801781

[B38] Zheng L. Ren R. Sun X. Zou Y. Shi Y. Di B. (2021). Discovery of a dual tubulin and poly(ADP-ribose) polymerase-1 inhibitor by structure-based pharmacophore modeling, virtual screening, molecular docking, and biological evaluation. J. Med. Chem. 64, 15702–15715. 10.1021/acs.jmedchem.1c00932 34670362

[B39] Zhou H. Cai Y. Liu D. Li M. Sha Y. Zhang W. (2018). Pharmacological or transcriptional inhibition of both HDAC1 and 2 leads to cell cycle blockage and apoptosis via p21Waf1/Cip1 and p19INK4d upregulation in hepatocellular carcinoma. Cell Prolif. 51, e12447. 10.1111/cpr.12447 29484736 PMC6528930

[B40] Zhou L. Zhang Y. Chen S. Kmieciak M. Leng Y. Lin H. (2015). A regimen combining the Wee1 inhibitor AZD1775 with HDAC inhibitors targets human acute myeloid leukemia cells harboring various genetic mutations. Leukemia 29, 807–818. 10.1038/leu.2014.296 25283841 PMC4387110

[B41] Zhou Y. Zou Y. Yang M. Mei S. Liu X. Han H. (2022). Highly potent, selective, biostable, and cell-permeable cyclic d-peptide for dual-targeting therapy of lung cancer. J. Am. Chem. Soc. 144, 7117–7128. 10.1021/jacs.1c12075 35417174

