# Supplementary material for: Discovery of novel and highly potent dual-targeting PKMYT1/HDAC2 inhibitors for hepatocellular carcinoma through structure-based virtual screening and biological evaluation
Source: Front Pharmacol. 2024 Nov 15;15:1491497. doi: 10.3389/fphar.2024.1491497 (PMC11604427; doi:10.3389/fphar.2024.1491497)
Supplement: Supplementary file 1 [file Table1.DOCX]

**Supplementary Material**

**Discovery of novel and highly potent dual-targeting PKMYT1/HDAC2 inhibitors for hepatocellular carcinoma through structure-based virtual screening and biological evaluation**

1. **Supplementary Figures and Tables**

**1.1 Supplementary tables**

**Supplementary Table S1.** Selectivity testing of PKHD-5 on a panel of 63 kinases.

| **Target** | **IC_50_ (μM)** | **Target** | **IC_50_ (μM)** | **Target** | **IC_50_ (μM)** |
| --- | --- | --- | --- | --- | --- |
| ABL1 | > 50 | FES | > 50 | LTK | > 50 |
| ABL2 | > 50 | FGFR1 | > 50 | LYN | > 50 |
| AXL | > 50 | FGFR2 | > 50 | MERTK | > 50 |
| BLK | > 50 | FGFR3 | > 50 | MET | > 50 |
| BMX | > 50 | FGFR4 | > 50 | MST1R | > 50 |
| BTK | > 50 | FGR | > 50 | MUSK | > 50 |
| CSF1R | > 50 | FRK | > 50 | NTRK1 | > 50 |
| PLK1 | > 50 | FYN | > 50 | NTRK2 | > 50 |
| DDR1 | > 50 | PIM1 | > 50 | NTRK3 | > 50 |
| DDR2 | > 50 | RAF1 | > 50 | PDGFRA | > 50 |
| ALK | > 50 | ROS1 | > 50 | PDGFRB | > 50 |
| EPHA1 | > 50 | ZAK | > 50 | PTK2 | > 50 |
| EPHA2 | > 50 | TYRO3 | > 50 | CDK1 | > 50 |
| EPHA3 | > 50 | YES1 | > 50 | ERBB4 | > 50 |
| EPHA4 | > 50 | ZAP70 | > 50 | KIT | > 50 |
| EPHA5 | > 50 | HCK | > 50 | KDR | > 50 |
| EPHA6 | > 50 | IGF1R | > 50 | ERBB2 | > 50 |
| EPHA7 | > 50 | INSR | > 50 | JAK3 | > 50 |
| EPHA8 | > 50 | INSRR | > 50 | PLK2 | > 50 |
| EPHB1 | > 50 | ITK | > 50 | PLK3 | > 50 |
| EPHB2 | > 50 | JAK1 | > 50 | JAK2 | > 50 |

**Supplementary Table S2.** Pharmacokinetic parameters for PKHD-5 in mice*^a^*.

| **Name** | **Route** | **T_1/2_ (h)** | **C_max_ (nmol/mL)** | **AUC (h·nmol /mL)** | **F (%)** |
| --- | --- | --- | --- | --- | --- |
| PKHD-5 | IP | 4.75 | 3264 | 15041 | 82 |
|  | PO | 3.93 | 2915 | 12163 | 66 |

*^a^*T_1/2_, elimination half-life; C_max_, maximum plasma concentration; AUC, area under the drug-time curve; *F,* bioavailability.
